# Burden of mental disorders by gender in Pakistan: analysis of Global Burden of Disease Study data for 1990–2019

**DOI:** 10.1192/bjb.2023.76

**Published:** 2024-12

**Authors:** Mohsin Hassan Alvi, Tehmina Ashraf, Farah Naz, Asif Sardar, Akbar Ullah, Anita Patel, Tayyeba Kiran, Anil Gumber, Nusrat Husain

**Affiliations:** 1 Pakistan Institute of Living and Learning, Rawalpindi, Pakistan; 2 University of Manchester, Manchester, UK; 3 Anita Patel Health Economics Consulting Ltd, London, UK; 4 Sheffield Hallam University, Sheffield, UK

**Keywords:** Burden, mental disorder, reproductive age, gender, Pakistan

## Abstract

**Aims and method:**

We aimed to examine the burden of mental disorders in Pakistan over the past three decades. We used the crude data of disability-adjusted life-years (DALYs) obtained from the Global Burden of Disease Study database (1990–2019) to represent burden. Data were retrieved on 26 January 2021. Data for adults of reproductive age (aged 15–49 years) were analysed to discuss and interpret the disease burden. An analysis was conducted on total DALYs separately for the genders for ten mental disorders reported in Pakistan.

**Results:**

DALYs increased drastically with the onset of reproductive age. Depressive disorder was the most reported mental disorder, contributing 3.13% (95% CI 2.25–4.24) of total DALYs, and varied significantly between genders: females 3.89% (95% CI 2.73–5.29) versus males 2.37% (95% CI 1.62–3.25).

**Clinical implications:**

A nationwide high-quality epidemiological surveillance system should be implemented to monitor mental disorders and offer culturally appropriate preventive services.

Mental, neurological and substance use disorders (MNS) are common, highly disabling and significantly associated with premature death. These disorders are the second leading contributor to total disability-adjusted life-years (DALYs) among males (48.1%) and the leading contributor among females (51.9%).^1^ According to the World Health Organization (WHO), around 13% of patients are affected by mental or neurological disorders across the globe.^2^ Among MNS, common mental disorders are one of the leading contributors to burden of disease globally,^3^ responsible for 22.9% of global years lived with disability (YLDs) and 7.4% of global DALYs, and hence are the leading cause of YLDs and the fifth leading contributor to DALYs.^4^ Eighty per cent of people with mental disorders live in low- and middle-income countries (LMICs).^3^ Moreover, mental disorders are associated with huge economic costs, more than the cost of long-term physical health conditions such as diabetes or cancer.^5^ According to the WHO, mental disorders result in economic loss of more than US$1 trillion per year.^6^ These disorders have been prioritised in global health policy and well-being is now included as one of the United Nation's sustainable development goals.^7^ Given the high DALYs associated with common mental disorders there is a great need to establish a national surveillance system in Pakistan to address the knowledge gap and monitor these illnesses; such a system should include national mental health surveys based on paper and pencil and telephone interviews, personal interviews and health and nutrition examination, pregnancy risk assessment, ambulatory medical care, hospital discharge and admission, economic conditions and poverty.^8^

In South Asians, mental disorders account for 12.2% of total health problems.^9^ A review of studies from the South Asian region suggested high prevalence rates for mental disorders, including depression, anxiety, mood disorders, suicidal behaviour and self-harm, schizophrenia, substance use disorders, neurodevelopmental disorders, dementia and other mental health conditions.^10^ The prevalence of mental disorders in Pakistan is as high as 10%, affecting approximately 20 million Pakistanis, and mental illness is associated with huge economic burden.^11^ Because of financial difficulties faced by most families, these disorders are difficult to manage and treat.^12^

Mental health is deeply rooted in the social, cultural, religious, spiritual, historical and holistic aspects of human lives.^13^ The risk of developing mental disorders is high among the poor, homeless, the unemployed, persons with low educational status, migrants and refugees, and indigenous populations.^14^ Evidence from Pakistan indicates that the risk factors for developing mental disorders are gender (women are at higher risk),^15^ poverty,^16,17^ domestic violence,^17^ adverse childhood experiences,^18,19^ lack of social support, stressful life events^18,20^ and low educational status.^21^ Furthermore, restrictions in terms of lockdown and social distancing in response to the COVID-19 pandemic have led to economic recession, which is reflected in an increased rate of mental health conditions, including self-harm and suicide.^22^

Despite the high prevalence of mental disorders, evidence on their economic burden in Pakistan is limited.^23^ Therefore, the purpose of this study was to use data from the Global Burden of Diseases, Injuries and Risk Factors Study 2019 (GBD 2019)^24^ to assess the risk factors associated with different mental disorders in adults of reproductive age and to further examine burden of disease in Pakistan from 1990 to 2019 in the context of health economics modelling.

Pakistan has one of the highest mental illness rates in the world.^25^ According to WHO estimates, 24 million people in Pakistan require mental healthcare.^26^ Unfortunately, the country has only 0.19 psychiatrists for every 100 000 people.^27^ Highlighting the estimated mental health burden will advocate to policymakers to include the discussion in national health agenda. GBD is the most comprehensive database that reports mental disorders that are not reported in national reports or elsewhere.

The word ‘burden’ has been used in this study to reflect the technical language commonly used by the authors of GBD studies. There is no intention to express any negative connotation towards people experiencing mental health difficulties.

## Method

### Data sources

We analysed the GBD 2019 data-set,^24^ which is a collaborative, comprehensive research study conducted by a global network of more than 3600 researchers from more than 145 countries. GBD 2019 measured morbidity and mortality from 369 diseases and injuries, analysing 87 risk factors in 204 countries and territories.^24^ GBD 2019 used all available data for mortality, population, fertility, cause of death, incidence, prevalence and other epidemiological measures. To harmonise heterogeneous data and use all possible sources of data, GBD 2019 used a wide variety of statistical modelling methods to build estimates for all outcomes of interest across 204 countries and territories, including Pakistan. GBD 2019 generated a complete set of estimates for cause-specific mortality, years of life lost (YLLs), YLDs and DALYs from 1990 to 2019.

Mental disorders were discussed in GBD 2019 on the basis of the clinical diagnostic criteria from DSM-IV-TR^28^ or ICD-10.^29^ YLL refers to premature death in relation to the expected lifespan, measured in years.^30^ YLDs are calculated from the product of prevalence and weightage of disability for mental health condition with adjustments for comorbidities. DALYs are the sum of premature mortality (YLLs) and loss of productivity (YLDs).^4^

**Fig. 1:**
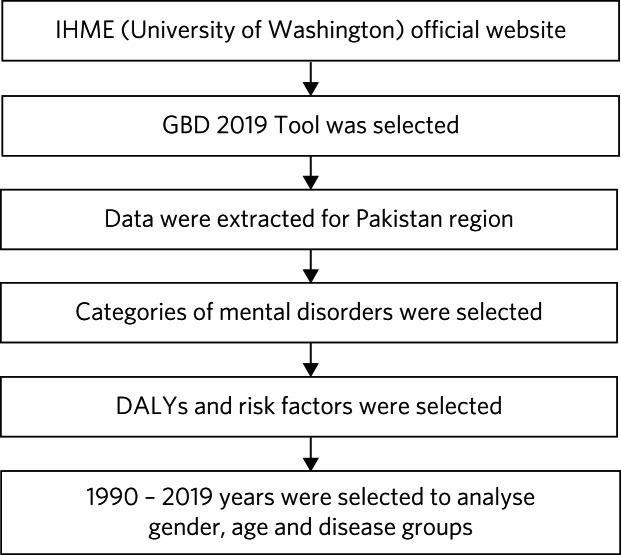
Data extraction flowchart. IHME, Institute for Health Metrics and Evaluation; GBD 2019, Global Burden of Diseases, Injuries and Risk Factors Study 2019; DALYs, disability-adjusted life-years.

For this study, we downloaded GBD 2019 statistics from the freely accessible resources on the Global Health Data Exchange (GHDx) (ghdx.healthdata.org/) and the GBD Results Tool (vizhub.healthdata.org/gbd-results/) with additional insights taken from corresponding data visualisations (vizhub.healthdata.org/gbd-compare/). These resources are hosted by the Institute of Health Metrics and Evaluation at the University of Washington. Specifically, we obtained GBD summary results (deaths, DALYs, YLLs and YLDs) for all causes of mental illness at all levels between 1990 and 2019 via the GBD Results Tool (Fig. 1).

**Fig. 2:**
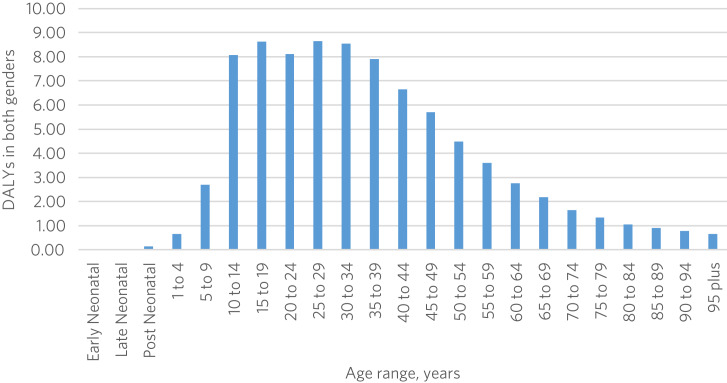
Age distribution of the population suffering with mental disorder in Pakistan, 2019.

The downloaded data for DALYs from GBD 2019 were put into our analysis. All categories of mental disorder gathered by GBD 2019 were selected. We focused on adults of both genders of reproductive age (aged 15–49 years) because we estimated that a large proportion of total disability is associated with this age group (Fig. 2). We gathered crude annual data for Pakistan for the full GBD 2019 study period (1990 to 2019). Data on YLLs and mortality were not considered in this study. Hence, almost all the proportion of DALYs has been contributed by YLDs.

### Analysis

Data were cleaned to remove typographical errors and duplicate values and manually checked to confirm authenticity prior to using them for further statistical analyses. Data were presented in tables and graphs, alongside relevant descriptive statistics. Analyses based on a previous study^31^ were carried out, with the current situation in Pakistan kept under consideration. The categories of mental disorders presented in the GBD 2019 tool were reported, along with subcategories. The gender-specific prevalence of each mental disorder and DALY rates in 2019 for Pakistan were reported. The prevalence rates of mental disorders from 1990 to 2019 for both the genders were reported, along with a gender comparison of change in DALY rate for Pakistan. The DALYs for specific mental disorders that were attributable to potential risk factors (such as bullying victimisation, intimate partner violence, childhood sexual abuse and lead exposure) in 2019 were also presented. The age-standardised rates were based on the GBD global reference population. In total, 18 estimates were reported, with 95% confidence intervals. Charts were supported by bar charts and pie charts for better understanding of results.

### Ethics statement

Data gathered from the Institute for Health Metrics and Evaluation's GBD 2019 data-set were presented fairly in analysis. No amendments or alterations were made to crude data, although data were checked for errors and duplication before analysis. The authors do not claim the ownership of data. All the tables and graphs presented in this paper were prepared by the authors. The economic outcomes of the data were disseminated honestly and are accessible to the lay public for awareness, critical appraisal and further research.

## Results

The greatest contribution to DALYs due to mental disorders in Pakistan was from depressive disorders (3.13%, 95% CI 2.25–4.24) and major depressive disorder (2.57%, 95% CI 1.78–3.53), followed by anxiety disorders (1.7%, 95% CI 1.21–2.30), schizophrenia (0.97%, 95% CI 0.68–1.25), dysthymia (0.57%, 95% CI 0.39–0.79) and bipolar disorder (0.46%, 95% CI 0.29–0.68); attention-deficit hyperactivity disorder (ADHD; 0.039%, 95% CI 0.022–0.063) and anorexia nervosa (0.06%, 95% CI 0.036–0.096) made the least contribution to DALYs. Depressive disorders and major depressive disorder contributed to DALYs over the 20-year period for both males and females, but females showed a relatively higher burden than males. Anxiety was the second highest contributor to mental illness. The contribution of depressive disorders and eating disorders to total DALYs was substantially higher in females than in males, whereas the contribution of autism spectrum disorders and ADHD was significantly higher in males than in females (Table 1).

**Table 1:** Proportion of disability-adjusted life-years (DALYs) attributable to each mental disorder in Pakistan, 2019

Causes	Both genders, % (95% CI)	Males, % (95% CI)	Females, % (95% CI)
Depressive disorder	3.13 (2.25–4.24)	2.37 (1.62–3.25)	3.89 (2.73–5.29)
Major depressive disorder	2.57 (1.78–3.53)	1.89 (1.23–2.68)	3.25 (2.20–4.53)
Dysthymia	0.57 (0.39–0.79)	0.49 (0.32–0.69)	0.64 (0.44–0.92)
Anxiety	1.70 (1.21–2.30)	1.49 (1.03–2.09)	1.92 (1.34–2.65)
Schizophrenia	0.97 (0.68–1.25)	1.14 (0.80–1.49)	0.81 (0.57–1.07)
Bipolar disorder	0.46 (0.29–0.68)	0.50 (0.30–0.75)	0.42 (0.26–0.62)
Conduct disorder	0.21 (0.12–0.34)	0.30 (0.17–0.48)	0.13 (0.07–0.24)
Intellectual disability	0.37 (0.20–0.59)	0.47 (0.25–0.75)	0.28 (0.15–0.45)
Autism spectrum disorder	0.19 (0.13–0.26)	0.28 (0.19–0.39)	0.10 (0.07–0.14)
Eating disorder	0.21 (0.13–0.30)	0.16 (0.10–0.24)	0.25 (0.16–0.37)
Anorexia nervosa	0.06 (0.04–0.10)	0.03 (0.02–0.06)	0.09 (0.05–0.14)
Bulimia nervosa	0.15 (0.09–0.22)	0.13 (0.07–0.20)	0.17 (0.10–0.25)
ADHD	0.04 (0.02–0.06)	0.06 (0.03–0.10)	0.02 (0.01–0.03)
Other mental disorder	0.45 (0.29–0.65)	0.55 (0.35–0.83)	0.35 (0.22–0.53)

ADHD, attention-deficit hyperactivity disorder.

**Fig. 3:**
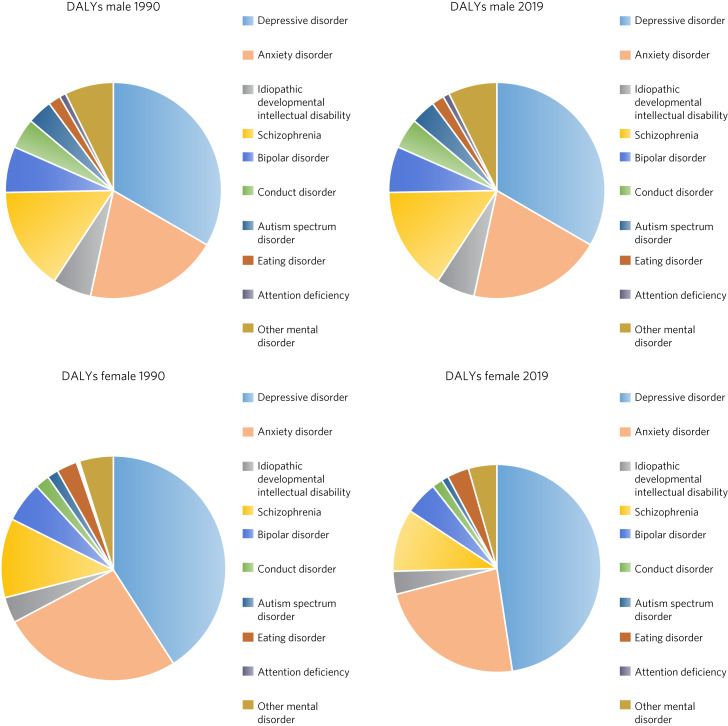
Comparison of disability-adjusted life-years (DALYs) by gender in Pakistan for 1990 and 2019.

Childhood sexual abuse, intimate partner violence and bullying were the factors contributing to depressive and anxiety disorders. Although childhood sexual abuse and bullying victimisation also contributed to DALYs, intimate partner violence was the major risk factor, accounting for 11% of DALYs (Table 2). The proportion of DALYs contributed by childhood sexual abuse was 5.3% in females, which was significantly higher than in males (3.97%). Bullying victimisation accounted for 12.2% of DALYs for anxiety disorder. A large proportion of DALYs for developmental intellectual disability (65.85%) was attributable to lead exposure (Fig. 3).

**Table 2:** Risk factors for mental disorders in Pakistan, 2019

		Proportion of total DALYs attributable to each risk factor, % (95% CI)		
Mental disorder	Risk factor	Both genders	Males	Females
Idiopathic developmental disorder	Lead exposure	65.85 (35.2–84.19)	66.57 (35.91–84.85)	64.69 (33.65–83.16)
Anxiety disorder	Bullying victimisation	12.20 (3.19–24.45)	14.07 (3.73–28.06)	10.81 (2.85–21.89)
Depressive disorder	Intimate partner violence	11.00 (0.07–22.39)	Not applicable	17.49 (0.10–35.76)
Depressive disorder	Bullying victimisation	7.06 (1.14–14.68)	8.15 (1.65–16.91)	6.42 (1.23–13.40)
Depressive disorder	Childhood sexual abuse	4.80 (2.51–7.92)	3.97 (1.78–7.23)	5.30 (1.78–7.23)

DALYs, disability-adjusted life-years.

## Discussion

The analysis of data from GBD 2019 shows that the overall proportion of DALYs in Pakistan due to mental disorders increased between 1990 and 2019. The pattern and magnitude of mental disorders changed over that period with respect to gender. Several risk factors have been identified that are likely to contribute to the burden of mental disorders in Pakistan. Findings also highlighted that young and middle-aged adults (aged 15–49 years) are experiencing a higher burden of mental disorders than children and older adults.

### Depression and anxiety disorders

According to the WHO, more than 322 million people globally are living with depression and rates increased between 2005 to 2015.^32^ The prevalence of depression is particularly high in South Asia (27%). The WHO has ranked depression as the single largest contributor to global disability, and anxiety disorders are ranked sixth.^32^ Similar trends have been reflected in the current study, with depression as the greatest contributor to DALYs in Pakistan, followed by anxiety disorders. These findings highlight the need for universal screening for depression and developing and testing culturally appropriate psychosocial interventions.^33^ Such interventions are hugely important, considering that depression is a major risk factor for self-harm and suicide.^32^

### Gender

The burden of mental disorders was found to be higher in females than in males. This is consistent with global trends.^32^ Women globally have reported significantly poorer health than men in terms of health-related quality of life, depression and psychological distress.^34^ Several risk factors, such as women's low self-esteem, high rates of stressful life events, interpersonal violence, discrimination and lack of gender equality, make women more vulnerable to developing mental health problems.^35^ Women during the reproductive years are particularly vulnerable to developing mental disorders; a study in Nigeria reported that prevalence of mental disorders (depression and anxiety) is 60.7% among women of reproductive age.^36^ Evidence supports that both genes and gene–environment interactions contribute to the risk of depression in a gender-specific manner.^36^ Therefore, strategies to incorporate mental health into reproductive health services may contribute to reducing this high burden. A series paper in the *Lancet* reported that Pakistan had the highest rate of stillbirths (43.1 per 1000 total births) across the globe.^37^ There is also a need to promote public health interventions to target women who have gynaecological conditions and who have experienced miscarriage/stillbirth to effectively reduce the burden of mental health problems in this age group.^38^

### Adverse childhood experiences and intimate partner violence

In the current study childhood sexual abuse, intimate partner violence and bullying were the major factors contributing to depressive and anxiety disorders. Studies have shown that childhood trauma is associated with a range of adverse physical, psychological and social outcomes.^38^ Individuals with a history of childhood abuse are significantly more likely to develop major depressive disorder in adulthood.^39^ A meta-analysis suggests that approximately 46% of individuals with depression reported childhood abuse, which is also associated with an earlier onset, a chronic course and treatment resistance for depression.^40^ Recent evidence from Pakistan shows that the majority of women with depression (58%) had had an adverse childhood experience such as home violence and neglect.^19,41^ Bullying is another type of childhood traumatic experience that is a major psychosocial concern because of its implications for subsequent behavioural and mental maladjustments over time. Individuals with history of childhood bullying are at increased risk of anxiety and depressive symptoms as well as future bullying and victimisation.^42^ These findings highlight the need for translational research that could potentially help in exploring strategies that might play a role in dealing with the impact of childhood abuse and bullying, particularly in individuals with major depressive disorder. Individualised culturally appropriate evidence-based psychosocial interventions that specifically address this subgroup are needed.^43^ Digitally delivered psychosocial interventions such as TechMotherCare^44^ may help to improve the mental health of the general population.^45^

Intimate partner violence has also been reported as a risk factor for mental disorders (depression and anxiety) in this study. Intimate partner violence is a significant human rights issue and global health concern, particularly in LMICs, with numerous detrimental outcomes, including intimate partner homicide^46^ and suicide.^47^ According to the WHO's global estimates, on average, up to 852 million women (almost 1 in 3 women) aged 15 years or older in 2018 had experienced intimate partner violence at least once in their lifetime.^48^ The prevalence of intimate partner violence in South Asia is the highest, at 35%.^45^ The Pakistan Demographic and Health Survey for 2012–2013 reported that 34% of ever-married women had experienced spousal physical, sexual or emotional violence, and 7% of women who had ever been pregnant had experienced violence during pregnancy.^49^ In addition, the containment measures introduced by the government as a result of the COVID-19 pandemic led to exacerbation of psychological adversities, including increased rates of intimate partner violence.^50^ Reducing gender-based violence is an indicator of one of the United Nation's sustainable development goals.^51^ Research conducted in high-income countries shows that mental health interventions may reduce the risk for victimisation by treating mental health problems and empowering women.^52^ However, the evidence from LMICs such as Pakistan is limited.^53^

### Addressing the burden of mental disorders: information and intervention

Mental health is being neglected in national surveys of Pakistan,^54–56^ resulting in limited robust data as well as contradictions in reports. Providing national level high-quality information is a first milestone towards strengthening the mental health surveillance system. This system will play a vital role in monitoring and reporting the national mental health burden and how much resources are needed to cover this burden. The accurate prevalence of diagnosable mental, neurological and substance use disorders (MNS) in Pakistan remains unclear^57^ because of limited high-quality research evidence on mental health.^58^ In addition to limited research, there are also problems at service delivery level because of limited trained staff to meet the needs of the population, contributing to the treatment gap. One of the recent initiatives to improve the health and well-being of people in Pakistan is the ‘President's Programme to Promote Mental Health of Pakistanis’ launched in 2019.^59^ Based on the recommendations made by the Lancet Commission on Global Mental Health, the programme emphasises the potential role of early-life interventions that promote mental health and well-being and prevent MNS, and it calls for implementation of the WHO Thinking Healthy Programme for mothers^60^ and the WHO School Mental Health Programme adapted for Pakistan.^61^ A similar programme, learning through play (LTP) plus CBT, is currently being implemented across Karachi city, which has a population of 23 million.^41^ The National Health Vision of Pakistan 2016–2025 is intended to focus on improving health facilities, with a particular focus on communicable and non-communicable diseases, including mental illness.^62^ The health minister of Sindh province of Pakistan has taken an initiative to implement a culturally adapted improving access to psychological therapy progamme (IAPT-PK) in Dadu and Qambar Shahdadkot districts, which are most affected by recent floods, in order to treat mental health conditions among the population.^63^

### Limitations

The limitations of the GBD methods, including those for estimation of MNS, have been documented in published literature. They include underestimation of the total uncertainty for DALYs; time difference in the reporting of health data by countries and their subsequent incorporation into the GBD estimations; and the scarcity of data for particular locations, leading to wider uncertainty intervals.^51^ A major limitation of this secondary analysis for Pakistan is that accurate and reliable population-level data on the prevalence of MNS are very limited across Pakistan, which may have created unknown biases in estimates reported in this paper. An estimation of the prevalence of common mental disorders has been reported in this study using data gathered from the GBD data-set that might vary from actual figures as the context is highly dynamic. The GBD estimation of burden of MNS relies on severity distribution data from high-income countries, which may not be a true reflection of the distribution in LMICs such as Pakistan.

## Data Availability

The data that support the findings of this study are openly available on the Global Health Data Exchange at vizhub.healthdata.org/gbd-results.

## About the authors

**Mohsin Hassan Alvi** is a research fellow, Department of Research, Pakistan Institute of Living and Learning, Rawalpindi, Pakistan. **Tehmina Ashraf** is a research assistant, Department of Research, Pakistan Institute of Living and Learning, Rawalpindi, Pakistan. **Farah Naz** is a research fellow, Department of Research, Pakistan Institute of Living and Learning, Rawalpindi, Pakistan. **Asif Sardar** is a research fellow, Department of Research, Pakistan Institute of Living and Learning, Rawalpindi, Pakistan. **Akbar Ullah** is a research fellow, Division of Population Health, Health Services Research & Primary Care, University of Manchester, Manchester, UK. **Anita Patel** is Director of Anita Patel Health Economics Consulting Ltd, London, UK. **Tayyeba Kiran** is Assistant Director, Department of Research, Pakistan Institute of Living and Learning, Rawalpindi, Pakistan. **Anil Gumber** is Principal Health Economist, Faculty of Health and Wellbeing, Sheffield Hallam University, Sheffield, UK. **Nusrat Husain** is Professor of Psychiatry and Director of Research for Global Mental Health, Division of Psychology & Mental Health, University of Manchester, Manchester, UK.
